# Understanding integrated care: a comprehensive conceptual framework based on the integrative functions of primary care

**DOI:** 10.5334/ijic.886

**Published:** 2013-03-22

**Authors:** Pim P. Valentijn, Sanneke M. Schepman, Wilfrid Opheij, Marc A. Bruijnzeels

**Affiliations:** Jan van Es Institute, Netherlands Expert Centre Integrated Primary Care, The Netherlands; NIVEL, Netherlands Institute for Health Services Research, The Netherlands; Twynstra Gudde, Consultants and Managers, The Netherlands; Jan van Es Institute, Netherlands Expert Centre Integrated Primary Care, The Netherlands

**Keywords:** primary care, integrated care, collaboration, fragmentation, care coordination

## Abstract

**Introduction:**

Primary care has a central role in integrating care within a health system. However, conceptual ambiguity regarding integrated care hampers a systematic understanding. This paper proposes a conceptual framework that combines the concepts of primary care and integrated care, in order to understand the complexity of integrated care.

**Methods:**

The search method involved a combination of electronic database searches, hand searches of reference lists (snowball method) and contacting researchers in the field. The process of synthesizing the literature was iterative, to relate the concepts of primary care and integrated care. First, we identified the general principles of primary care and integrated care. Second, we connected the dimensions of integrated care and the principles of primary care. Finally, to improve content validity we held several meetings with researchers in the field to develop and refine our conceptual framework.

**Results:**

The conceptual framework combines the functions of primary care with the dimensions of integrated care. Person-focused and population-based care serve as guiding principles for achieving integration across the care continuum. Integration plays complementary roles on the micro (clinical integration), meso (professional and organisational integration) and macro (system integration) level. Functional and normative integration ensure connectivity between the levels.

**Discussion:**

The presented conceptual framework is a first step to achieve a better understanding of the inter-relationships among the dimensions of integrated care from a primary care perspective.

## Introduction

The aging population and the growing prevalence of chronic conditions increases the healthcare costs and utilization of many high income countries [1, 2]. Integrated health systems have been promoted as a means to improve access, quality and continuity of services in a more efficient way, especially for people with complex needs (e.g., multiple morbidities) [3–6]. Primary health care (as a set of principles and policies) and primary care (as a set of clinical functions) are considered as the corner stones of any health system (throughout this paper both ‘primary care’ and ‘primary health care’ are used interchangeably and referred to as ‘primary care’) [7–9]. Health systems built on the principles of primary care (first contact, continuous, comprehensive, and coordinated care) achieve better health and greater equity in health than systems with a specialty care orientation [9, 10]. The philosophy of primary care goes beyond the realm of healthcare and requires inter-sectorial linkages between health and social policies [7, 8]. Hence, the definition of primary care assumes an integrated view with the rest of the health system. However, in many high income countries integration of services is hampered by the fragmented supply of health and social services as a result of specialisation, differentiation, segmentation and decentralisation [5, 8, 11]. Fragmentation results in suboptimal care, higher cost due to duplication and poor quality of care [5]. In the Netherlands the Primary focus program aims to stimulate integration (both within primary care and between primary care and other health and social service sectors) by funding 70 collaboration initiatives [12]. To discover the critical factors that hamper or facilitate integration starting from a primary care perspective, the development process of these collaboration initiatives is monitored. A conceptual framework is needed to make systematic and comparable descriptions of these initiatives. However, the concept of integrated care is ambiguous, since it is often used as an umbrella term that differs in underlying scope and value [4, 5, 13–15]. This lack of conceptual clarity hampers systematic understanding and hence the envision, design, delivering, management and evaluation of integrated care. There seems to be a growing need for a conceptual framework to understand the complex phenomenon of integrated care and to guide empirical research [13, 16]. The aim of this paper is to develop a conceptual framework for integrated care from a primary care perspective. In this paper we use the definition of integrated care of Leutz (1999) [17] and the definition of primary care as stated in the Alma-Ata Declaration [7], see Table 1. This paper proposes a conceptual framework that can contribute to a better understanding of the concept of integrated care from a primary care perspective.

## Methods

The framework was developed through an iterative process of: (1) a narrative literature review, and (2)\xc2\xa0group meetings and expert panels to synthesise the literature.

### Literature search

We conducted a narrative literature review to identify existing conceptual and theoretical concepts regarding primary care and integrated care. The literature search involved a combination of electronic database searches, hand searches of reference lists of papers and contacting researchers in the field. We focused on the three concepts of the Primary focus program: (1) primary care; (2) integrated care; and (3) collaboration. The preliminary search started in the electronic databases Medline/PubMed, Cochrane Library and Google Scholar using the search terms ‘primary care’ and/or ‘integrated care’ combined with ‘cooperation’ or ‘collaboration’. The following ‘MeSH’ terms were used to broaden the search in Medline/PubMed: ‘Primary Health Care’ and ‘Delivery of Health Care, Integrated’. We included journal articles, books and book chapters written in English that reported conceptual and theoretical concepts related to primary care, integrated care and collaboration. Potentially relevant references were further obtained from the retrieved publications and by contacting researchers in the field (snowball method).

### Building the framework

The process of synthesising the literature was iterative. The lead author reviewed the literature, and catalogued the different conceptual and theoretical concepts. The research team chose the key features of primary care as a base on which to develop a more comprehensive framework. Next, we connected the ambiguous concepts of integrated care with the key features of primary care into a first draft of the framework. To improve the content validity of the framework we discussed it with seven researchers in the field of integrated care and primary care. During six meetings of approximately one hour a discussion was held on the synthesis of the essential elements of primary care and integrated care. Based on these discussions we refined the framework.

## Results

To construct the conceptual framework we used 50 articles obtained by our search. Eighteen were found by direct searches in databases and 25 by using the snowball method. We used 12 articles to identify the key elements of primary care and 34 articles to describe the key elements of integrated care. Table 2 shows the key elements of primary care and integrated care that we identified with our literature search.

In the following sections, we will outline the pillars of our framework: (1) the key elements of primary care, (2) the dimensions of integrated care, and (3) the combination of the key elements of primary care and integrated care.

## Integrative function of primary care

Primary care as stated in the declaration of Alma-Ata in 1978 is a strategy of public health (e.g., a health policy at the macro level) derived from a social model of health, making it possible to distribute health services equitably across populations [7], see Table 1. This philosophy contains a number of different concepts, namely: equity on the basis of need, first level of care usually encountered by the population, a political movement, a philosophy underpinning service delivery and a broad inter-sectorial collaboration in dealing with community problems. Taken together, a broad public health policy encompassing a wide range of integration functions and goals. The functions of primary care (first-contact, continuous, comprehensive, and coordinated care, see Table 2) [10, 18] make it possible to accomplish the integrated philosophy that is envisaged in the Alma Ata Declaration. Together these functions make primary care the starting point from where to improve and integrate care. The most evident function ‘first contact’ gives primary care a central position within the health system. It refers to the directly accessible ambulatory care for each new problem at all times and at close proximity of its users. The second function ‘continuity’ refers to the experienced coherence of care over time that addresses the need and preferences of people. Hereby the personal experience is essential, as continuity is what people experience. The third function ‘comprehensiveness’ refers to an array of services tailored to the needs of the population served. These services comprise curative, rehabilative and supportive care as well as health promotion and disease prevention. The fourth function ‘coordination’ means that people are referred both horizontally and vertically when services from other providers are needed. All together, these functions give primary care a central role in coordinating and integrating care.

### A person and population health-focused view

Enclosed in the functional conceptualisation of primary care is the person and population health-focused view. This holistic vision is expressed as person-focused and population-based care [7, 8, 10]. The first feature, person-focused care, reflects a bio-psychosocial perspective of health, as it acknowledges that health problems are not synonymous to biological terms, diagnoses or diseases [22]. It bridges the gap between medical and social problems as it acknowledges that diseases are simultaneously a medical, psychological and social problem [23]. Moreover, person-focused care is based on personal preferences, needs, and values (i.e., understanding the personal meaning of an illness). In contrast, a disease-focused view reflects a clinical professionals perspective, translating the needs of a person into distinct biological entities that exist alone and apart form a person [24–26]. The second feature, population-based care, attempts to address all health-related needs in a defined population. In this view services should be based on the needs and health characteristics of a population (including political, economic, social, and environmental characteristics) to improve an equitable distribution of health (and well-being) in a population [10]. The need and equity focus of population-based care is especially important for socially disadvantaged subpopulations with higher burdens of morbidity [8]. Population-based care entails defining and categorizing populations according to their burden of morbidity. However, Western health systems are dominated by the paradigm of a disease-focused view, that neglects the underlying causes of health and well-being [27]. This view is dysfunctional in a population, because a growing number of patients suffer from chronic and overlapping health problems (e.g., multi-morbidity) [28]. Therefore, the person and population health-focused view is essential, as it recognizes that most health and social problems are inter-related. This is especially important in the context of integrated care as the person-focused and population-based perspective can link the health and social systems.

## Dimensions of integrated care

The second pillar in our conceptual model is the dimensions of integrated care. These dimensions are structured around the three levels where integration can take place: the macro (system) level, the meso (organisational) level and the micro (clinical) level [29]. We start with drawing the contours of an integrated system at the macro level and then continue to the meso and micro level using the integrative guiding principles of primary care: person-focused and population-based care.

### The macro level: system integration

**Figure 1.**
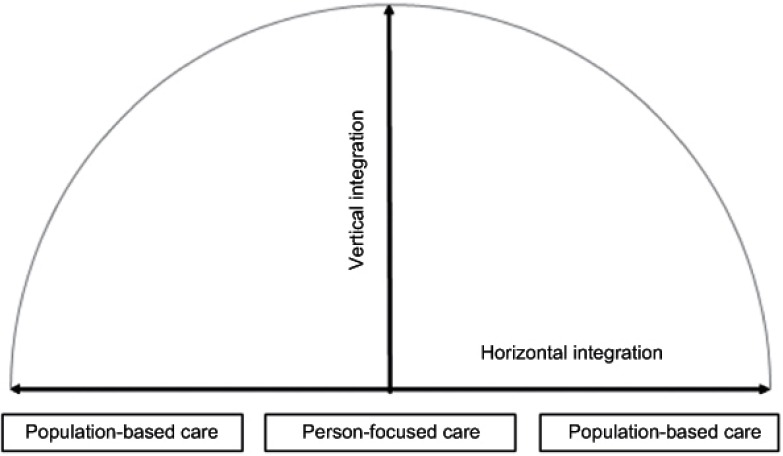
System integration.

At the macro level system integration is considered to enhance efficiency, quality of care, quality of life and consumer satisfaction [5, 6]. The integration of a health system is an holistic approach that puts the people’s needs at the heart of the system in order to meet the needs of the population served (note the similarity to the definition of primary care) [4, 6, 13]. System integration requires a tailor-made combination of structures, processes and techniques to fit the needs of people and populations across the continuum of care [4, 5]. However, the current specialisation in health systems (e.g., disease-focused medical interventions) causes fragmentation of services threatening the holistic perspective of primary care [11]. A resultant of the specialisation and fragmentation is vertical integration (see Table 2). Vertical integration is related to the idea that diseases are treated at different (vertical) levels of specialisation (i.e., disease-focused view). This involves the integration of care across sectors, e.g., integration of primary care services with secondary and tertiary care services. Contrary, horizontal integration is improving the overall health of people and populations (i.e., holistic-focused view) by peer-based and cross-sectorial collaboration [30]. Primary care and public health are characterized by horizontal integration to improve overall health [31]. The distinction between these integration mechanisms is important, because they require different techniques to be achieved and are based on different theories of change and leadership [30]. Nevertheless, both vertical and horizontal integration are needed to counteract the fragmentation of services in a health system [14, 16]. Incorporating vertical and horizontal integration can improve the provision of continuous, comprehensive, and coordinated services across the entire care continuum. In other words, partnerships across traditional organisational and professional boundaries are needed in order to improve the efficiency and quality of a system [32, 33]. In an integrated system these partnerships can pass through the boundaries of the ‘cure’ and ‘care’ sector to provide a real continuum of care to people and populations. Figure 1 shows an integrated health system with the person-focused care and population-based care perspective as the foundation for system integration. They serve as guiding principles within a system, which requires simultaneously horizontal (x-axis) and vertical integration (y-axis).

### Meso level: organisational integration

One of the most discussed forms of integration is organisational integration, conceptualised at the meso level of a health care system [21]. Organisational integration refers to the extent that services are produced and delivered in a linked-up fashion. Inter-organisational relationships can improve quality, market share and efficiency; for example, by pooling the skills and expertise of the different organisations [3, 5, 16, 21, 34]. To deliver population-based care organisational integration is needed [16, 35]. The needs of a population require collective action of organisations across the entire care continuum (horizontal and vertical integration), as they have a collective responsibility for the health and well-being of a defined population. Especially in socially disadvantaged populations, such as those with large variations in wealth, education, culture and access to health care, the need for integration is high [5, 13]. However, the broad spectrum of organisations needed to assure good health in a population makes organisational integration complicated [5, 16]. For instance, health and social care organisations can differ distinctively in terms of culture, professional roles and responsibilities, and clinical or service approaches [13]. Furthermore, the differences in bureaucratic structures, levels of expertise, funding mechanisms and regulations can complicate organisational integration [36].

### Market, hierarchy and networks

**Figure 2.**
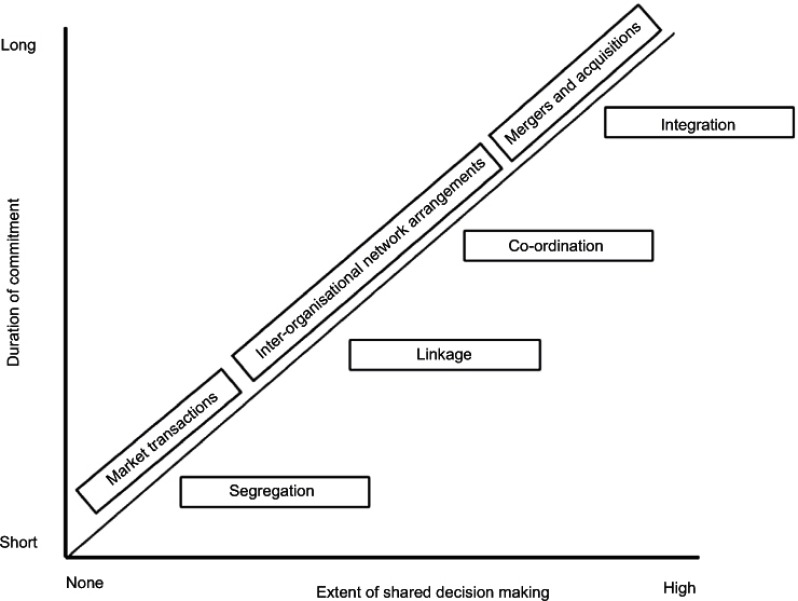
Continuum of inter-organisational integration. Source: Adapted from Gomes-Casseres (2003) [44] and Ahgren and Axelsson (2005) [41].

Organisational integration can be achieved through hierarchical governance structures or through market-based governance structures between organisations [37]. Markets are more flexible than hierarchies, but the commitment between the organisations is minimal compared to hierarchies. Alternative for hierarchical or market-based governance structures are network-like governance mechanisms, which means a more or less voluntary collaboration between organisations. They depend on relationships, mutual interests, and reputation and are less guided by a formal structure of authority [38]. Networks are considered as the golden mean which unite flexibility and commitment. Network-like partnerships are prevalent in health and social care [5, 16, 39, 40], as these arrangements are able to address the opposing demands of state regulation and market competition present in many Western health care systems. The extent of organisational integration is often expressed as a continuum, ranging from segregation to full integration [17, 41]. In a segregated situation every organisation is autonomous, with organisations functioning as independent entities. On the other hand, full integration contains hierarchical mechanisms of governance such as mergers and acquisitions. The intermediate levels of inter-organisational integration reflect the network-like governance mechanisms; linkage and coordination. The typology of ‘loose’ to ‘tight’ governance agreements is widespread in the literature [39, 42, 43]. Gomes-Casseres (2003) [44] describes a model that is similar to the continuum of organisational integration and ranges from market situations through inter-organisational network arrangements to mergers and acquisitions. His model states that the complexity of inter-organisational networks results from ambiguous shared decision-making and unclear duration of commitment. In Figure 2, the above-mentioned theories of organisational integration and inter-organisational arrangements are combined.

The left hand side of Figure 2 shows a segregated situation, where market competition leads to contractual relations between the organisations. In this scenario the duration of commitment and extent of shared decision-making is short-term as a result of the ‘invisible hand’ of market competition [37]. The right hand side shows a full integrated situation, a top-down coordination of organisations. In this scenario the duration of commitment and extent of shared decision-making is long-term as a result of the ‘visible hand’ of a management hierarchy [37]. The central part of Figure 2 shows a network mode of integration, and explains the complexity of this type of arrangements due to the continuous tension between flexibility and commitment. Within a network management cannot exercise authority or legitimate power because each organisation retains its autonomy (reflected by shared decision-making) [39]. This requires the involved organisations to continuously negotiate and assess the outcomes of the collaboration, resulting in an uncertain and changing environment (reflected by duration of commitment) [20].

Organisational integration in the field of primary care is often done according to a network mode [45]. This is, as most primary care organisations are not market oriented and many of them are not part of a common hierarchy [16]. However, these complex network arrangements require effective mechanisms of accountability and governance. Governance structures should align the different independent organisations and coordinate their interdependencies [6, 20]. To summarise, organisational integration contains several types of inter-organisational relationships on the meso level of a system that provide comprehensive services across the care continuum. Organisational integration is defined as follows: Inter-organisational relationships (e.g., contracting, strategic alliances, knowledge networks, mergers), including common governance mechanisms, to deliver comprehensive services to a defined population.

### Meso level: professional integration

Professional integration refers to partnerships between professionals both within (intra) and between (inter) organisations [5], and is conceptualised on the meso-level of a health system [21]. These partnerships can be characterised as forms of vertical and/or horizontal integration. Professionals have a collective responsibility to provide a continuous, comprehensive, and coordinated continuum of care to a population [6, 21, 32, 46, 47]. Especially in populations with a growing burden of disease, professionals from a range of disciplines and sectors have to take shared responsibility for the integration of services to assure good health and well-being. Integration led by professionals creates combined responsibilities for commissioning services and promotes shared accountability, problem solving and decision-making to achieve optimal health and well-being in a defined population [35]. As a consequence of this approach, the professional autonomy is affected and the traditional hierarchy and clear defined roles are blurred [48]. Professional integration can be achieved through a variety of arrangements from virtually integrated professional networks to fully integrated organisations [17, 49]. The extent of professional integration is expressed as a continuum similar to that of organisational integration (with fragmentation, linkage, coordination and full integration) [48]. Professional integration in primary care is traditionally characterised by network like arrangements, that create poor conditions for shared accountability [45]. Appropriate financing and regulation incentives can stimulate this [6, 32, 45, 50]. Besides the fiscal and clinical dimensions of accountability it is unclear what other types of accountability are required. However, a lack of shared language and divergent healing paradigms can make professional integration difficult. Clarity about roles, responsibilities and principles of altruism, ethics, respect and communication seem to be crucial to overcome these difficulties [51]. The challenge is to stimulate accountable entrepreneurial professionals, while at the same time leaving sufficient freedom for different professional healing paradigms.

We define professional integration as follows: Inter-professional partnerships based on shared competences, roles, responsibilities and accountability to deliver a comprehensive continuum of care to a defined population.

### Micro level: clinical integration

At the micro level of a health system, clinical integration refers to the coherence in the primary process of care delivery to individual patients [21]. Clinical integration refers to the extent to which patient care services are coordinated across various professional, institutional and sectorial boundaries in a system [32]. Kodner [5] equates clinical integration with service integration: “coordination of services and the integration of care in a single process across time, place and discipline” (p. 11). In practice, clinical integration tends to be a disease-focused approach rather than a person-focused approach [52]. For instance, most tools and instruments of clinical integration are based on narrow, disease-oriented medical interventions [10, 52, 53]. The limits of clinical guidelines are increasingly recognized, particularly when the broader health context is involved, e.g., by chronic multi-morbidities [54]. This is particularly relevant for socially disadvantaged people (and populations) whose needs span a number of service areas. In practice, clinical integration requires a person-focused perspective to improve someone’s overall well-being and not focus solely on a particular condition. Professionals have to take proper account of the needs of individuals, so that services provided are matched to their needs. This also encloses the important aspect of the patient as a co-creator in the care process; with shared responsibility between the professional and the person to find a common ground on clinical management [55, 56]. Emphasis should be placed on a person’s needs, with people coordinating their own care whenever possible [14]. In other words, clinical integration based on a person-focused care perspective, can facilitate the continuous, comprehensive, and coordinated delivery of services at an individual level.

Our definition of clinical integration is as follows: The coordination of person-focused care in a single process across time, place and discipline.

### Linking the micro, meso and macro level: functional integration

Functional integration supports clinical, professional, organisational and system integration [57]. It refers to mechanisms by which financing, information, and management modalities are linked to add the greatest overall value to the system [32]. Functional integration includes the coordination of key support functions such as financial management, human resources, strategic planning, information management and quality improvement [20, 35]. It involves shared policies and practices for support functions across partnerships between different actors within a system. However, functional integration does not mean more centralisation or standardisation [35]. Functional integration should be a flexible approach in order to enable partnerships to adapt to the constantly changing environment (e.g., population needs). One of the most important aspects of functional integration is the linking of the financial, management, and information systems around the primary process of service delivery (clinical integration) [35, 58]. These linked systems can support and coordinate policy-makers (system integration), managers (organisational integration), professionals (professional integration) and patients (clinical integration) in their accountability and shared decision-making in (inter-sectorial) partnerships. To sum, functional integration supports and links the clinical (micro-level), professional and the organisational integration (meso-level) dimensions within a system (macro-level).

Functional integration is defined as follows: Key support functions and activities (i.e., financial, management and information systems) structured around the primary process of service delivery, to coordinate and support accountability and decision-making between organisations and professionals to add overall value to the system.

### Linking the micro, meso and macro level: normative integration

Another integration dimension that achieves connectivity and also spans the micro, meso and macro level in a system is known as normative integration [5, 19, 20]. It is a less tangible but essential feature to facilitate inter-sectorial collaboration and ensure consistency between all the levels of an integrated system. Veil and Hébert [58] define normative integration as: “ensuring coherency between the actors’ systems of value, service-organization methods, and the clinical system” (p. 76). Integration is to a large extent shaped by and based on professional behaviour and attitudes [34, 41, 59]. Informal coordination mechanisms based on shared values, culture, and goals across individuals, professionals and organisations are considered as essential. Person-focused and population-based care are important social norms, that should guide behaviour within a health system. In the involvement of various actors different frames of reference need to be combined to improve the health of a population. The clashing of cultures (e.g., between medical and non-medical professionals) is one of the reasons why many integration efforts fail [6, 45]. A clear mission and vision that reflects the needs of the local population is considered a critical success factor for population-based care [32, 60]. Mutual shared goals and an integrative culture are necessary at all levels of an integrated system, and can be created by leadership [6]. Particularly at the professional and management level, leadership plays an important role in propagating an integrated approach [6, 20, 58].

Normative integration can provide a common frame of reference that binds together all the levels of an integrated system. Normative integration is defined as follows: The development and maintenance of a common frame of reference (i.e., shared mission, vision, values and culture) between organisations, professional groups and individuals.

## Combining primary care and integrated care

**Figure 3.**
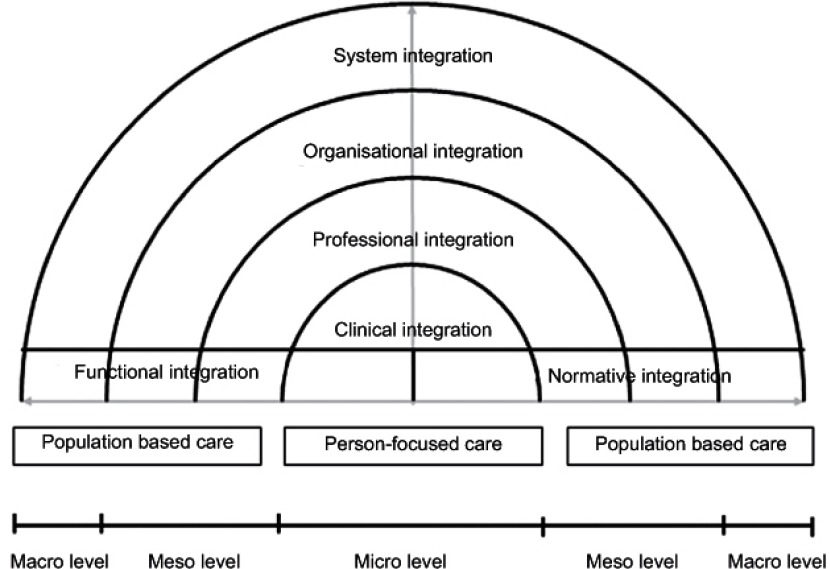
Conceptual framework for integrated care based on the integrative functions of primary care.

Figure 3 shows our conceptual framework that combines the primary care and integrated care literature into a holistic picture. The core value of primary care is the integration of the biomedical, psychological and social dimensions of health and well-being, expressed in our conceptual framework as person-focused and population-based care. The person-focused and population-based care perspectives provide a foundation upon which the entire conceptual framework rests. They serve as guiding principles for achieving better coordination of services across the entire care continuum. The integrative functions of primary care (first contact, continuous, comprehensive, and coordinated care) are incorporated implicit in the dimensions of integrated care. We make a distinction between the levels of care when focusing on integration. At the macro level system integration puts the individual needs at the heart of the system in order to meet the needs of the population. That is because system integration incorporates the notion that what is best for individuals within a population is best for the population. This holistic view requires simultaneous horizontal and vertical integration to improve the overall health and well-being of individuals and the population. Our framework is therefore visualised as a concentric circle, with the person-focused perspective at the centre. Integration at the meso level emphasises a population-based approach, requiring professional and organisational integration to facilitate the continuous, comprehensive, and coordinated delivery of services to a defined population. At the micro level clinical integration highlights the person-focused perspective, ensuring that service users experience continuous care. Health professionals have to take proper account of the needs of individuals, so that the services provided are matched (both horizontally and vertically) to their needs. This may mean that integration may be pursued at the meso and macro level, when services from other providers or organisations are needed. Finally, functional and normative integration spans the micro, meso and macro level and ensures connectivity within a system.

## Discussion

This paper contributes to the conceptualisation of integrated care from a primary care perspective. We constructed a framework to understand the complex phenomenon of integrated care. This means a simplification of reality which helps to better understand the complex interactions of integrated care [16]. We suggest that integration has to be pursued at different levels within a system to facilitate the continuous, comprehensive, and coordinated delivery of services to individuals and populations. How these integration levels interact will vary according to the specific context in which they develop. There are several directions for further research grounded in our new framework. First, the model provides further guidance to study the preferred directions of integration: Is it for instance a ‘bottom-up’ (clinical), ‘top-down’ (system) or two-sided (bottom-up and top-down) approach as specified by Kodner and Spreeuwenberg [13]. Second, the framework provides directions to identify the optimal scenario for integration and the contribution of the different integration mechanisms. For instance, our model in combination with the work of Leutz [17] and Ahgren and colleagues [41] can be used to discover the extent of integration at all integration levels in conjunction. However, there are some methodological challenges that arise from our conceptualisation. First, evidence-based knowledge about integration is hampered by the lack of standardised, validated tools and indicators to measure integration [61, 62]. For instance, most available evidence is based on small pilots, what makes it difficult to generalise these findings [63]. Second, there is often a lack of information regarding the validity and reliability of measurement tools [61, 62]. The inter-sectorial nature of integrated care and primary care requires a comprehensive mixed method approach that can be applied across multiple settings [64, 65]. However, most literature on the measurement of integrated care contains a wide variety of concepts, methods and measurements [61]. More research is needed to build up evidence with validated measurement tools to evaluate integrated care initiatives in a more synergetic and analytic way. The conceptual framework presented in its current form is intended for further testing, refinement and development. As the conceptual framework is built on the theoretical concept of primary care, we invite further discussion on whether and how far the framework may apply in other integrated care settings (for example in specialty care or intramural settings). Ultimately, we hope to develop our framework as a tool for conducting analysis of integrated care initiatives to be used to test for causal relationships among the different integration levels. Thereafter the framework will be validated in the Primary focus program. We hope that our framework provides a comprehensive base for policy-makers, managers, professionals and other stakeholders to better understand the synergetic nature of integrated care.

## Conclusion

We conclude that to deliver integrated, person-focused, and population-based care, vertical- and horizontal integration through inter-sectorial partnerships across the health and social service system is needed. Our conceptualization includes multiple dimensions of integration that play complementary roles on the micro (clinical integration), meso (professional- and organisational integration) and macro (system integration) level to deliver comprehensive services that address the needs of people and populations. Functional and normative integration can ensure connectivity of all the levels of an system.

## Figures and Tables

**Table 1.**
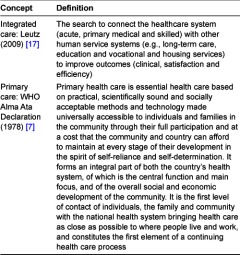
Definitions of integrated care and primary care.

**Table 2.**
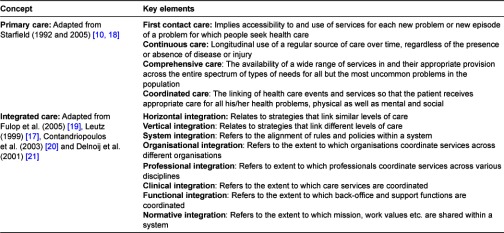
Key elements of primary care and integrated care.
